# Artificial intelligence (AI) versus expert: A comparison of left ventricular outflow tract velocity time integral (LVOT‐VTI) assessment between ICU doctors and an AI tool

**DOI:** 10.1002/acm2.13724

**Published:** 2022-07-11

**Authors:** Shanshan Zhai, Hui Wang, Lichao Sun, Bo Zhang, Feng Huo, Shuang Qiu, Xiaoqing Wu, Junyu Ma, Yina Wu, Jun Duan

**Affiliations:** ^1^ Department of Surgery Intensive Care Unit China–Japan Friendship Hospital Beijing China; ^2^ Department of Emergency Medicine China–Japan Friendship Hospital Beijing China; ^3^ Department of Ultrasound Medicine China–Japan Friendship Hospital Beijing China; ^4^ Department of Emergency Medicine, National Center for Children's Health, Beijing Children's Hospital Capital Medical University Beijing China; ^5^ Department of Intensive Care Unit The Sixth Clinical Medical College of Henan University of Traditional Chinese Medicine Zhumadian Henan Province 463000 China

**Keywords:** artificial intelligence, critical care, left ventricular outflow tract velocity time integral, point of care ultrasound training

## Abstract

**Purpose:**

The application of point of care ultrasound (PoCUS) in medical education is a relatively new course. There are still great differences in the existence, quantity, provision, and depth of bedside ultrasound education. The left ventricular outflow tract velocity time integral (LVOT‐VTI) has been successfully used in several studies as a parameter for hemodynamic management of critically ill patients, especially in the evaluation of fluid responsiveness. While LVOT‐VTI has been broadly used, valuable applications using artificial intelligence (AI) in PoCUS is still limited. We aimed to identify the degree of correlation between auto LVOT‐VTI and the manual LVOT‐VTI acquired by PoCUS trained ICU doctors.

**Methods:**

Among the 58 ICU doctors who attended PoCUS training from 1 September 2019 to 30 November 2020, 46 ICU doctors who trained for more than 3 months were enrolled. At the end of PoCUS training, each of the enrolled ICU doctors acquired echocardiography parameters of a new ICU patient in 2 h after new patient was admitted. One of the two bedside expert sonographers would take standard echocardiogram of new ICU patients within 24 h. For ICU doctors, manual LVOT‐VTI was obtained for reference and auto LVOT‐VTI was calculated instantly by using an AI software tool. Based on the image quality of the auto LVOT‐VTI, ICU patients was separated into ideal group (*n* = 31) and average group (*n* = 15).

**Results:**

Left ventricular end‐diastolic dimension (LVEDd, *p* = 0.1028), left ventricular ejection fraction (LVEF, *p* = 0.3251), left atrial dimension (LA‐d, *p* = 0.0962), left ventricular E/A ratio (*p* = 0.160), left ventricular wall motion (*p* = 0.317) and pericardial effusion (*p* = 1) had no significant difference between trained ICU doctors and expert sonographer. ICU patients in average group had greater sequential organ failure assessment (SOFA) score (7.33 ± 1.58 vs. 4.09 ± 0.57, *p* = 0.022) and lactic acid (3.67 ± 0.86 mmol/L vs. 1.46 ± 0.12 mmol/L, *p* = 0.0009) with greater value of LVEDd (51.93 ± 1.07 vs. 47.57 ± 0.89, *p* = 0.0053), LA‐d (39.06 ± 1.47 vs. 35.22 ± 0.98, *p* = 0.0334) and percentage of decreased wall motion (*p* = 0.0166) than ideal group. There were no significant differences of δLVOT‐VTI (|manual LVOT‐VTI – auto LVOT‐VTI|/manual VTI*100%) between the two groups (8.8% ± 1.3% vs. 10% ± 2%, *p* = 0.6517). Statistically, significant correlations between manual LVOT‐VTI and auto LVOT‐VTI were present in the ideal group (R^2^ = 0.815, *p* = 0.00) and average group (R^2^ = 0.741, *p* = 0.00).

**Conclusions:**

ICU doctors could achieve the satisfied level of expertise as expert sonographers after 3 months of PoCUS training. Nearly two thirds of the enrolled ICU doctors could obtain the ideal view and one third of them could acquire the average view. ICU patients with higher SOFA scores and lactic acid were less likely to acquire the ideal view. Manual and auto LVOT‐VTI had statistically significant agreement in both ideal and average groups. Auto LVOT‐VTI in ideal view was more relevant with the manual LVOT‐VTI than the average view. AI might provide real‐time guidance among novice operators who lack expertise to acquire the ideal standard view.

## 1 INTRODUCTION

Recent studies^1^, ^2^ reported lower complication rates and mortality after point of care ultrasound (PoCUS)‐based guidance compared with standard patient management, one of the most well‐validated PoCUS applications was related to predict fluid responsiveness.^3^, ^4^, ^5^, ^6^ The main limitation of fluid responsiveness tests is that it requires a direct measurement of cardiac output in real time to capture the maximum effects of the infused fluid or passive leg raising (PLR) test,^7^ which occur within seconds and vanish after 1 min in some patients.^8^ The left ventricular outflow tract velocity time integral (LVOT‐VTI) is a non‐invasive, simple, rapid, repeatable parameter for assessing cardiac output when measured by experienced operators.^9^

At the present time, most ICU physicians are not yet trained in PoCUS program.^10^ Even though the 2‐3‐day live learning courses with an online learning curriculum and final assessment is one of the most reputable and rigorous certifications, PoCUS in clinical management after the short course is still a challenge for most ICU doctors who lack the nuances of ventricular function, valvular assessment, and hemodynamic measurements.^11^ To ensure maintenance of high‐quality and reproducible scanning for ICU doctors, our center provided a long course PoCUS program.

Furthermore, interrater expertise variability has been recognized as a potential source of inadequate diagnostics and therapeutic decisions. The misuse or misinterpretation of focused echocardiography by novice or inexperienced practitioners has the potential for harm.^12^ Ostensibly, acquiring, retaining, and sharing data that are augmented by artificial intelligence (AI) will mitigate inter‐operator variability and afford safer clinical practice.^13^ Recently, application of AI in LVOT‐VTI have been deployed to reduce operator‐dependent differences and save time with the utilization of the auto LVOT‐VTI software^13^, ^14^, ^15^

The objective of this study was to compare echocardiography parameters obtained by ICU doctors who finished our long‐course PoCUS program to those of expert sonographer, as well as to identify the degree of correlation between auto LVOT‐VTI and the manual LVOT‐VTI acquired by ICU doctors.

## 2 METHOD

This perspective study was approved by the research ethics committee of China–Japan Friendship Hospital(2018‐49‐K38). We have recruited advanced ICU doctors (*n* = 58) for PoCUS training from 1 September 2019 to 30 November 2020. ICU doctors who trained for more than 3 months were included in our study while those who trained less than 3 months were excluded. ICU doctors enrolled were separated into four to six teams in order to make the ratio of instructors to doctors lower than 1:5. We adopted a new model of “Three Progressive Levels” for hands‐on sessions: first level was teaching and practicing on simulation manikins, second level was performing scans of healthy volunteers and third level was examining the real ICU patients. All of these hands‐on learning was supervised by experienced mentors. ICU doctors were required to take images proficiently within 5 min on simulation manikins and healthy individuals before they could take echocardiogram of actual ICU patients. After 3 months of PoCUS training, each ICU doctor was asked to acquire echocardiography parameters of a new ICU patient in 2 h after the patient was admitted to our department. In addition to standard echocardiogram measurements, ICU doctors were required to obtain the manual LVOT‐VTI and auto LVOT‐VTI (calculated by the AI software tool). There were two bedside expert sonographers on duty by turns in our hospital and both of them were included in our study. The bedside expert sonographer on duty would take standard echocardiogram of new ICU patients within 24 h. Since the ICU patients were admitted without any plan, one of those two expert sonographers took bedside echocardiography for new ICU patients randomly. The clinical characteristics of ICU patients, including the main reason for admittance to ICU, acute physiology and chronic health enquiry (APACHE‐II) score, sequential organ failure assessment (SOFA) score, invasive treatments, central venous pressure (CVP), lactic acid, N‐terminal pro‐B type natriuretic peptide (NT‐proBNP), fluid balance in first 24 h, and in‐hospital mortality were collected as well.

**FIGURE 1:**
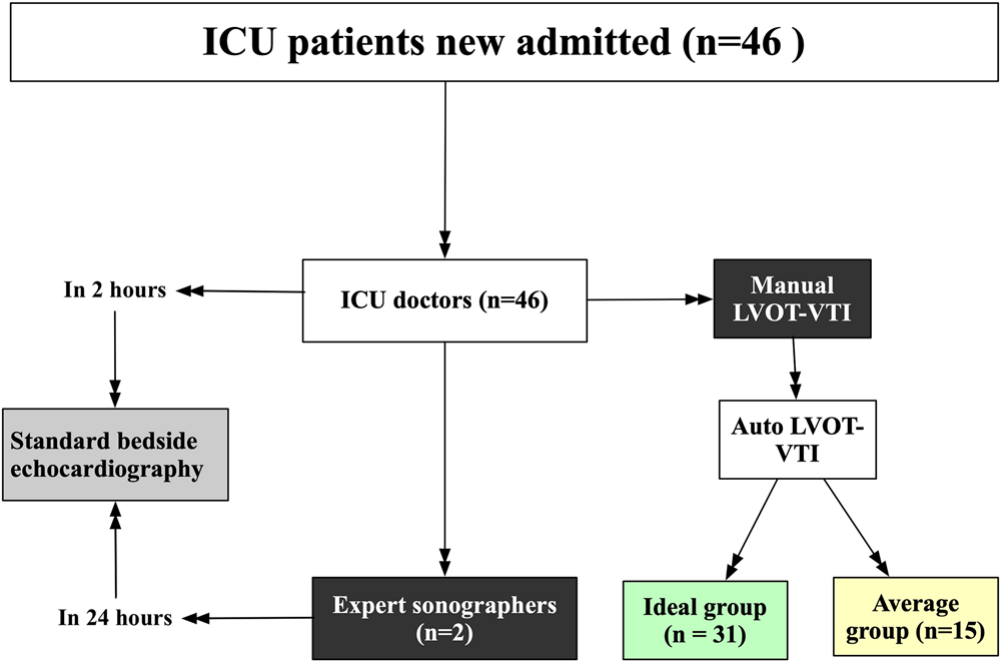
Diagram of our study. Left side of figure, standard bedside echocardiography examinations of ICU patients newly admitted acquired by ICU doctors in 2 h and by expert sonographers in 24 h. Right side of figure, in addition to standard measurements, ICU doctors acquired manual LVOT‐VTI and auto LVOT‐VTI as well. New ICU patients were separated into ideal group and average group on the basis of the image quality of auto LVOT‐VTI (green color indicated better image quality than yellow color)

For expert sonographers on duty in our hospital, the total number of echocardiography examinations per day was more than 20, including almost 10 bedside echocardiography of newborn babies that occupied much time of the experts. Thus, the heavy workload makes it impossible to create a “gold standard” of the LVOT‐VTI measured by experts at the same time when ICU doctors performed bedside echocardiography, and LVOT‐VTI varies a lot during 24 h of critically ill ICU patients. To reduce the variations between manual and auto LVOT‐VTI that change over time, we applied a two‐step design for our study (Figure 1). First step was the comparison of standard bedside echocardiography measurements between the ICU doctors and expert sonographers to test the hypothesis that trained ICU doctors were able to obtain high scan quality of image as good as those expert sonographers. Only if the assumption of first step was verified, manual LVOT‐VTI could be a reliable reference for auto LVOT‐VTI calculated by AI tool for ICU doctors. In order to reduce time and rapid fluid induced variations of LVOT‐VTI, the manual and auto LVOT‐VTI were acquired by ICU doctors at the same time when there was no rapid fluid infusion for the patients. On the basis of the image quality of auto LVOT‐VTI, new ICU patients were separated into ideal group and average group.

**FIGURE 2:**
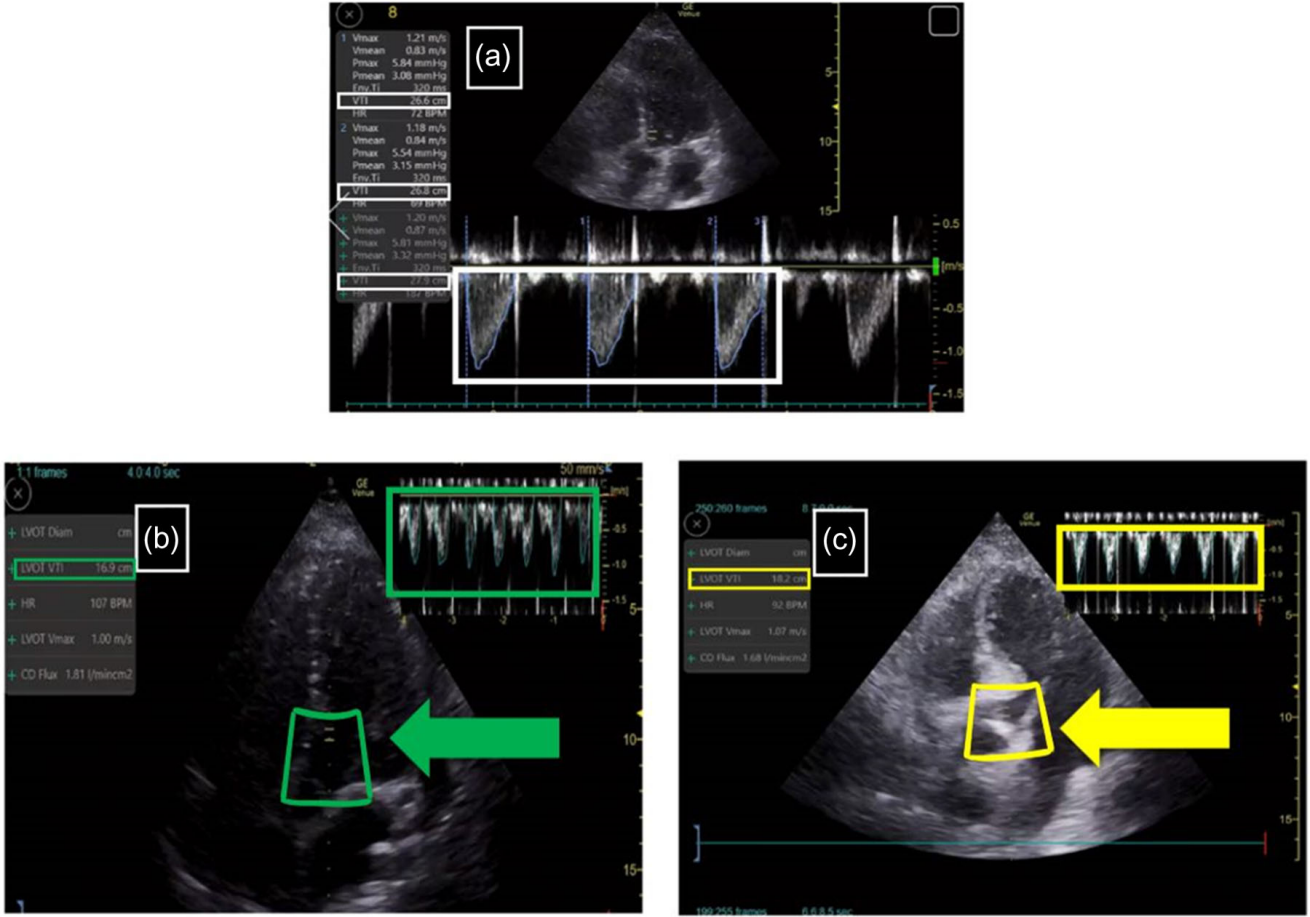
Manual LVOT‐VTI and auto LVOT‐VTI acquired by ICU doctors. (2a) Manual LVOT‐VTI measurements by ICU doctors in apical five‐chamber view, the large white box showed three cycles of manual LVOT‐VTI and the small white box indicated the exact value of each manual LVOT‐VTI. (2b) AI‐VTI tool screen layout of ideal view, the left green box displayed the auto LVOT‐VTI value calculated by AI software instantly, the middle green box of ROI and the green arrow emphasized the ideal quality of the image, the green box in the upper right corner contained all the cycles that AI calculated. (2c) AI‐VTI tool screen layout of average view, the left yellow box displayed the AI‐calculated auto LVOT‐VTI, the middle yellow box of ROI and the yellow arrow indicated the average quality of the image, the left box in the upper right corner contained all the cycles that AI calculated

In order to assess the scan quality of the ICU doctors, PoCUS parameters of ICU doctors were compared with expert sonographers. The bedside standard echocardiography report contained left ventricular end‐diastolic dimension (LVEDd), left ventricular ejection fraction (LVEF), left atrial dimension (LA‐d) left ventricular wall motion, left ventricular inflow/atrial‐systolic peak velocity (E/A ratio), right ventricular dimension (RV‐d) and pericardial effusion. For ICU doctors, LVOT‐VTI was obtained by placing a pulsed‐wave Doppler sample volume in the LVOT immediately proximal to the aortic valve in the anteriorly angulated apical five‐chamber view and tracing the outer boundaries of the peak spectral Doppler signal to obtain LVOT‐VTI (Figure 2a). The average of those separate LVOT‐VTI measurements was obtained as a manual LVOT‐VTI. Following manual VTI measurements (manual LVOT‐VTI), a software generated (*Venue, GE Medical Systems Ultrasound & Privacy Care Diagnostic LLC, Wauwatosa, United States*) automated LVOT‐VTI measurement (auto LVOT‐VTI) was obtained by each participant. The Auto‐VTI tool can be used when scanning the patient with a 3Sc‐RS phased array probe, using five‐chamber view from the apical position. After automatically locating and positioning the region of interest (ROI) over the LVOT, the tool finds the aortic valve and places the gate at an optimal position on the image. The calculations are done on real time and the results are displayed in the results box. The quality indicator is represented by the color of the ROI placed by the system over the image, and varies between green/yellow/red to represent ideal (Figure 2b) / average (Figure 2c) /unacceptable image quality, respectively. We aimed to evaluate the accuracy and consistency of auto LVOT‐VTI with the manual LVOT‐VTI in the ideal group and average group. In terms of the relative difference between manual LVOT‐VTI and auto LVOT‐VTI for each patient, we defined that δLVOT‐VTI was equal to |manual LVOT‐VTI − auto LVOT‐VTI|/manual VTI*100%. Besides manual and auto LVOT‐VTI, δLVOT‐VTI, heart rate of new patients, and cardiac cycles for calculating the average manual and auto LVOT‐VTI were recorded as well.

### 2.1 Statistics

The continuous data were presented as the means ± SD and noncontinuous variables are expressed as frequencies. We compared categorical variables by Pearson's *Chi‐Square* test. The difference of enumeration data between trained ICU doctors and the expert sonographer was detected by paired Chi‐Square (McNemar–Bowker test). The differences of echocardiography continuous variables between trained ICU doctors and the expert sonographer were examined by paired *t*‐test. We used two‐sample *t‐*test for the differences of continuous variables between ideal group and average group. Manual LVOT‐VTI and auto LVOT‐VTI were compared by pair *t*‐test and correlations between manual LVOT‐VTI and auto LVOT‐VTI were analyzed by linear regression. Stata/MP 14.1 (Stata Corp, Lakeway, TX, USA) and SPSS 22.0 were used for calculations and illustrations. Tables were created using asdoc, a Stata program written by Shah.^16^ All tests were two‐sided, and *p* values  < 0.05 were considered statistically significant.

## 3 RESULTS

Among the 58 of advanced ICU doctors who participated in PoCUS program of our center, 46 of them who trained for more than 3 months were included in our study and 12 of them who trained less than 3 months were excluded. After 3 months of PoCUS training, each of the enrolled ICU doctors had recorded an echocardiography report of a new ICU patient. Two expert sonographers on duty took standard bedside echocardiography for those 46 ICU patients randomly. For trained ICU doctors, auto‐LVOT‐VTI and manual‐LVOT‐VTI were obtained by placing the probe at the same position in the apical five‐chamber view. Based on the image quality of auto‐LVOT‐VTI, the ICU patients were separated into the ideal group (*n* = 31) and average group (*n* = 15).

### 3.1 Echocardiography measurements between trained ICU doctors and expert sonographer

In the parasternal long‐axis view, LVEDd (48.99 ± 0.75 mm vs. 48.06 ± 0.84 mm, *p* = 0.1028), LVEF (61.58 ± 1.97% vs. 60.39 ± 1.39%, *p* = 0.3251) and LA‐d (36.47 ± 0.85 mm vs. 35.52 ± 0.90 mm, *p* = 0.0962) had no significant difference between trained ICU doctors and expert sonographer. In the parasternal short‐axis view, trained ICU doctors and expert sonographers reported that the proportions of left ventricular wall motion and pericardial effusion were comparable between the two groups (*p* = 0.317 and *p* = 1). In the apical four‐chamber view, no significant difference was discovered in left ventricular diastolic function between trained ICU doctors and expert sonographer (*p* = 0.160). However, trained ICU doctors reported larger RV‐d (37.02 ± 0.66 mm vs. 34.39 ± 0.75 mm, *p* = 0.0003) than the expert sonographer (Table 1).

**TABLE 1:** Echocardiography parameters between trained ICU doctors and expert sonographers

Parameters	Trained ICU doctors (n = 46)	Expert sonographers (n = 2)	p value
**LVEDd (mm)**	48.99 ± 0.75	48.06 ± 0.84	0.1028
**LVEF (%)**	61.58 ± 1.97	60.39 ± 1.39	0.3251
**LA‐d (mm)**	36.47 ± 0.85	35.52 ± 0.90	0.0962
**Ventricular wall motion** ^a^ **(*n*, %)**			0.317
**Normal**	35 (76.1%)	36 (78.3%)	
**Mild decrease**	5 (10.9%)	4 (8.7%)	
**Moderate decrease**	6 (13%)	6 (13%)	
**Pericardial effusion** ^a^ **(n, %)**			1
**Absent**	41 (89.1%)	43 (93.5%)	
**Mild effusion**	4 (8.7%)	3 (6.5%)	
**Moderate effusion**	1 (2.2%)	0	
**RV‐d (mm)**	37.02 ± 0.66	34.39 ± 0.75	0.0003
**E/A ratio (diastolic function)** ^a^ **(*n*, %)**			0.160
**Normal**	14 (30.4%)	18 (39.1%)	
**Impaired relaxation**	28 (60.9%)	25 (54.4%)	
**Restrictive filling**	4 (8.7%)	3 (6.5%)	

Values are expressed as mean ± SD or *n* (%). E/A ratio, left ventricular inflow /atrial‐systolic peak velocity; 1 < E/A ratio < 2, normal diastolic function; E/A ratio < 1, impaired relaxation; E/A ratio > 2, restrictive filling.

Abbreviations: LA‐d, left atrial dimension; LVEDd, left ventricular end‐diastolic dimension; LVEF, left ventricular ejection fraction; RV‐d, right ventricular dimension.

^a^ The differences of ventricular wall motion, pericardial effusion, and E/A ratio (diastolic function) between two groups was detected by paired Chi‐Square (McNemar–Bowker test).

### 3.2 Clinical characteristics and echocardiography parameters in the ideal group and average group

As shown in Table 2, the clinical characteristics of ICU patients between the ideal group (*n* = 31) and average group (*n* = 15) were analyzed. As for gender and age, no significant differences were detected between the two groups (male, 45.1% vs. 60%, *p* = 0.3454; age, 69.5 ± 2.6 vs. 64.5 ± 3.8, *p* = 0.291). The population distribution of ICU patients in ideal group was comparable to average group (*p* = 0.272), the most common reason for admit to ICU was scheduled surgery (51.6% vs. 40%, *p* = 0.4598) followed by emergency surgery (25.8% vs. 26.7%, *p* = 0.7673), sepsis shock (3.2% vs. 20%, *p* = 0.0584), acute pancreatitis (6.5% vs. 6.7%, *p* = 0.9779), acute heart failure (9.7% vs. 0, *p* = 0.5405), pulmonary embolism (3.2% vs. 0, *p* = 1), and acute kidney failure (0 vs. 6.7%, *p* = 0.3261). Although there was no statistical difference, the incidence of sepsis shock seemed to be a bit higher in average group than in ideal group (20% vs. 3.2%, *p* = 0.0584). Moreover, we found that patients in average group had greater SOFA score (7.33 ± 1.58 vs. 4.09±0.57, *p* = 0.022) and lactic acid (3.67 ± 0.86 mmol/L vs. 1.46 ± 0.12 mmol/L, *p* = 0.0009) than ideal group. APACHE II score (13.90 ± 1.08 vs. 16.86 ± 2.57, *p* = 0.216), CVP (7.75 ± 0.50 mmHg vs. 7.9 ± 0.84 mmHg, *p* = 0.872), NT‐proBNP (2265 ± 1136 pg/ml vs. 3619 ± 2213 pg/ml, *p* = 0.548), input in first 24 h (1990.1 ± 118.5 ml vs. 2053.5 ± 173.4 ml, *p* = 0.762), and output in first 24 h (1513.6 ± 124.7 ml vs. 1261.6 ± 171.6 ml, *p* = 0.248) had no significant difference between ideal group and average group. The rate of continuous renal replacement therapy (CRRT) was a bit higher in average group than ideal group (13.3% vs. 0%, *p* = 0.0376). It seemed that patients in average group were a bit more likely to die (40% vs. 22.6%) and adopt ventilation (60% vs. 38.7%) than ideal group, although without significant statistic differences (*p* = 0.3785 and *p* = 0.1742).

**TABLE 2:** Clinical characteristics and echocardiography parameters in the ideal group and average group

Variables	Total (*n* = 46)	Ideal group (*n* = 31)	Average group (*n* = 15)	*p*
**Clinical characteristics**				
**Male (*n*, %)**	23 (50%)	14 (45.1%)	9 (60%)	0.3454
**Age (years old)**	67.9 ± 2.2	69.5 ± 2.6	64.5 ± 3.8	0.291
**Reason for admit to ICU (*n*, %)**			0.272	
**Scheduled surgery**	22 (47.8%)	16 (51.6%)	6 (40%)	0.4598
**Emergency surgery**	12 (26.1%)	8 (25.8%)	4 (26.7%)	0.7673
**Sepsis shock**	4 (8.7%)	1 (3.2%)	3 (20%)	0.0584
**Acute pancreatitis**	3 (6.5%)	2 (6.5%)	1 (6.7%)	0.9779
**Acute heart failure**	3 (6.5%)	3 (9.7%)	0	0.5405
**Pulmonary embolism**	1 (2.2%)	1 (3.2%)	0	1
**Acute kidney failure**	1 (2.2%)	0	1 (6.7%)	0.3261
**APACHE II score**	14.87 ± 1.11	13.90 ± 1.08	16.86 ± 2.57	0.216
**SOFA score**	5.15 ± 0.67	4.09 ± 0.57	7.33 ± 1.58	0.022
**CVP (mmHg)**	7.8 ± 0.43	7.75 ± 0.50	7.9 ± 0.84	0.872
**Lactic acid (mmol/L)**	2.18 ± 0.32	1.46 ± 0.12	3.67 ± 0.86	0.0009
**NT‐proBNP (pg/ml)**	2707 ± 1042	2265 ± 1136	3619 ± 2213	0.548
**Input in first 24 h (ml)**	2010.7 ± 96.8	1990.1 ± 118.5	2053.5 ± 173.4	0.762
**Output in first 24 h (ml)**	1431.5 ± 101.4	1513.6 ± 124.7	1261.6 ± 171.6	0.248
**Invasive treatments (*n*, %)**				
**Ventilation**	15 (32.6%)	12 (38.7%)	9 (60%)	0.1742
**CRRT**	2 (4.3%)	0	2 (13.3%)	0.0376
**Mortality (*n*, %)**	13 (28.2%)	7 (22.6%)	6 (40%)	0.3785
**Echocardiography parameters**				
**LVEDd (mm)**	48.99 ± 0.75	47.57 ± 0.89	51.93 ± 1.07	0.0053
**LVEF (%)**	61.58 ± 1.97	64.03 ± 2.43	56.53 ± 3.02	0.0737
**LA‐d (mm)**	36.47 ± 0.85	35.22 ± 0.98	39.06 ± 1.47	0.0334
**Ventricular wall motion (*n*, %)**			0.0482	
**Normal**	35 (76.1%)	25 (80.6%)	10 (66.7%)	0.2974
**Mild decrease**	5 (10.9%)	1 (3.2%)	4 (26.7%)	0.0166
**Moderate decrease**	6 (13%)	5 (16.1%)	1 (6.7%)	0.3717
**Pericardial effusion (*n*, %)**			0.335	
**Absent**	41 (89.1%)	28 (90.3%)	13 (86.6%)	0.7088
**Mild effusion**	4 (8.7%)	3 (9.7%)	1 (6.7%)	0.7341
**Moderate effusion**	1 (2.2%)	0	1 (6.7%)	0.3261
**RV‐d (mm)**	37.02 ± 0.66	36.35 ± 0.82	38.6.74 ± 1.06	0.151
**E/A ratio (diastolic function) (*n*, %)**		0.6514		
**Normal**	14 (30.4%)	8 (25.8%)	6 (40%)	0.3267
**Impaired relaxation**	28 (60.9%)	20 (64.5%)	8 (53.3%)	0.4663
**Restrictive filling**	4 (8.7%)	3 (9.7%)	1 (6.7%0	0.7341
**Manual LVOT‐VTI (cm)**	19.79 ± 0.68*	19.82 ± 0.88^#^	19.73 ± 1.06ˆ	0.9482
**Auto LVOT‐VTI (cm)**	19.48 ± 1.71	19.77 ± 1.01	18.87 ± 1.29	0.6015
**δLVOT‐VTI (%)**	9 ± 1.2	8.8 ± 1.3	10 ± 2	0.6517
**Heart rate (beats/minute)**	84.47 ± 2.78	84.09 ± 2.93	85.26 ± 6.19	0.8467
**Manual cardiac cycles (*n*)**	2.91 ± 0.06	2.83 ± 0.08	3.07 ± 0.12	0.1239
**Auto cardiac cycles (*n*)**	4.06 ± 0.25	4.29 ± 0.29	3.57 ± 0.45	0.1845

Values are expressed as mean ± S D or *n* (%). δLVOT‐VTI = |manual LVOT‐VTI − auto LVOT‐VTI|/manual VTI*100%. Abbreviations: APACHE II score, acute physiology and chronic health enquiry score; CRRT, continuous renal replacement therapy; CVP, central venous pressure; E/A ratio, left ventricular inflow /atrial‐systolic peak velocity; LA‐d, left atrial dimension; LVEDd, left ventricular end‐diastolic dimension; LVEF, left ventricular ejection fraction; NT‐proBNP, N‐terminal pro‐B type natriuretic peptide; RV‐d, right ventricular dimension; SOFA score, sequential organ failure assessment score.

*Manual LVOT‐VTI versus auto‐LVOT‐VTI in total, paired *T*‐test, *p* = 0.3093; ^#^Manual LVOT‐VTI versus auto‐LVOT‐VTI in ideal group, paired *T*‐test, *p* = 0.8895; ^ˆ^Manual LVOT‐VTI versus auto‐LVOT‐VTI in average group, paired *T*‐test, *p* = 0.1588.

In terms of echocardiography parameters in both groups (Table 2), we discovered that patients in average group had larger LVEDd (51.93 ± 1.07 vs. 47.57 ± 0.89, *p* = 0.0053) and LA‐d (39.06 ± 1.47 vs. 35.22 ± 0.98, *p* = 0.0334) than ideal group. The patients in average group had more decrease (mild) of LV wall motion (26.7% vs. 3.2%, *p* = 0.0166) than ideal group. In view of the relative difference between manual LVOT‐VTI and auto LVOT‐VTI for each patient, there was no significant differences of δLVOT‐VTI (|manual LVOT‐VTI − auto LVOT‐VTI|/manual VTI*100%) between the two groups (8.8% ± 1.3% vs. 10% ± 2%, *p* = 0.6517). Mean values of manual LVOT‐VTI in ideal group and average group were 19.82 ± 0.88 cm and 19.73 ± 1.06 cm while mean values of auto‐LVOT‐VTI in both groups were 19.77 ± 1.01 cm vs. 18.87 ± 1.29 cm. No significant differences were discovered in pericardial effusion (*p* = 0.335) and left ventricular diastolic function (*p* = 0.6514) between ideal group and average group. The other variables including LVEF (64.03 ± 2.43% vs. 56.53 ± 3.02%, *p* = 0.0737), RV‐d (36.35 ± 0.82 mm vs. 38.6.74 ± 1.06 mm, *p* = 0.151), heart rates (84.09 ± 2.93 beats/minute vs. 85.26 ± 6.19 beats/minute, *p* = 0.8467), manual cardiac cycles (2.83 ± 0.08 vs. 3.07 ± 0.12, *p* = 0.1239), and auto cardiac cycles (4.29 ± 0.29 vs. 3.57 ± 0.45, *p* = 0.1845) were similar between the ideal group and average group.

**FIGURE 3:**
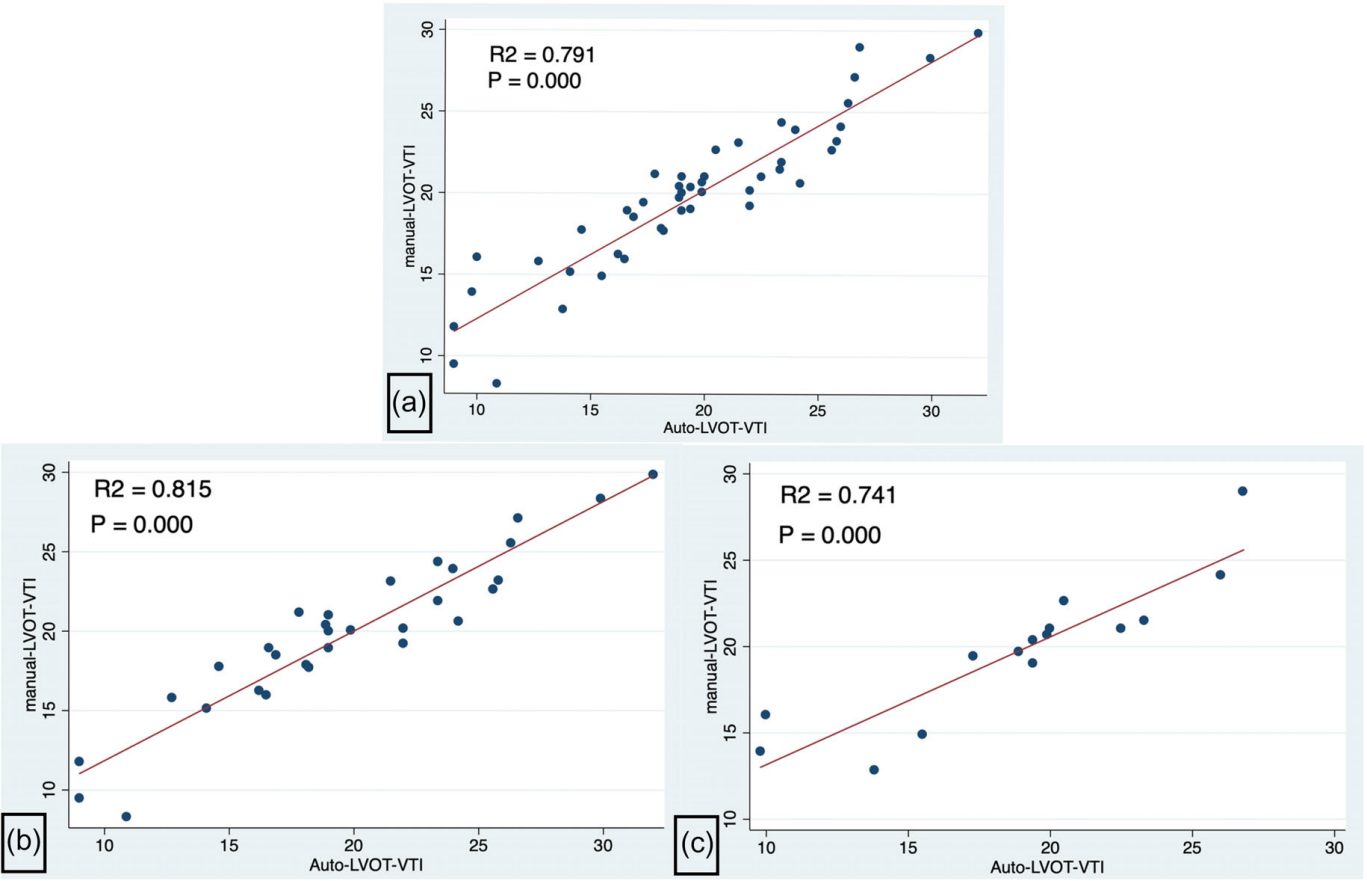
Correlations between manual LVOT‐VTI and auto‐LVOT‐VTI. (3a) Correlation between manual LVOT‐VTI and auto‐LVOT‐VTI in the total patients (R2 = 0.792, *p* = 0.000). (3b) Correlation between manual LVOT‐VTI and auto‐LVOT‐VTI in the ideal group (R2 = 0.815, *p* = 0.000). (3c) Correlation between manual LVOT‐VTI and auto‐LVOT‐VTI in the average group (R2 = 0.741, *p* = 0.000)

In order to evaluate the accuracy and consistency of auto‐LVOT‐VTI with the manual LVOT‐VTI, paired *T*‐tests between manual LVOT‐VTI and auto‐LVOT‐VTI showed no significant differences in total (*p* = 0.3093), the ideal group (*p* = 0.8895) and the average group (*p* = 0.1588). Statistically significant correlations between manual LVOT‐VTI and auto‐LVOT‐VTI were present in total (R2 = 0.792, *p* = 0.000), the ideal group (R2 = 0.815, *p* = 0.000) and average group (R2 = 0.741, *p* = 0.000) (Figure 3).

## 4 DISCUSSION

In the current study, we observed that there were no significant differences of standard echocardiography parameters between trained ICU doctors and expert sonographer. ICU patients of the average group had higher SOFA score and lactic acid with greater value of LVEDd and LA‐d than patients of ideal group. We also discovered that both the ideal and average auto LVOT‐VTI were comparable to manual LVOT‐VTI. In addition to that, we noticed that auto LVOT‐VTI in ideal view was more relevant with the manual LVOT‐VTI than the average view.

Previously, most PoCUS training studies (59.5%) had <20 trainees while a few researches enrolled more than 31 trainees.^17^ The total number of ICU doctors for PoCUS training in our study was 46 which was much higher than most reports. Compared with the 1‐2‐day PoCUS course, our 3‐month PoCUS program had a new model of “Three Progressive Levels” for hands‐on sessions supervised by experienced mentors. Our center had more than six experienced mentors and the ratio of instructors to trainees was one instructor for every four to five trainees in one group whereas the ratio was not reported in most studies.^17^ In learning a technical skill, the role of mentor is invaluable, as a mentor provides direction for deliberate practice, not just rote repetition.^11^ Deliberate step‐by‐step practices on simulation manikins, healthy volunteers, and real ICU patients allows for mastery of skills through focused and purposeful feedback. At the end of PoCUS training, the final assessment for ICU doctors was to record a formal bedside echocardiography report of a new ICU patient without supervision. To reduce the bias and make sure that ICU doctors took examination without knowing any detail about the patient's former echocardiography measurements, they were first required to complete the examination within 2 h. Moreover, to take objectivity and repeatability of echocardiography parameters into consideration, constant variables including LVEDd, LVEF, LA‐d, left ventricular wall motion and E/A ratio were selected as key indexes of parasternal long/short‐axis view and apical four/five‐chamber view. According to the similarity of parameters mentioned above, between trained ICU doctors and experts, we succeeded in verifying the hypothesis that ICU doctors could achieve the high level of expertise as expert sonographers after 3 months of PoCUS training. On the basis of reliable parasternal long/short‐axis view and apical four/five‐chamber view obtained by ICU doctors, manual LVOT‐VTI acquired by ICU doctors could be a “gold standard” reference for auto LVOT‐VTI.

Furthermore, among the clinical characteristics and echocardiography variables collected by ICU doctors, we found that there were several key factors that affected the quality of AI image, especially the state of illness of ICU patients newly admitted. As a surgery intensive care unit (SICU), nearly half of the ICU population were scheduled post‐operation patients. The rest of ICU population were consisted of emergency conditions such as emergency surgery, sepsis shock, acute pancreatitis, acute heart failure, acute pulmonary embolism, and acute kidney failure. The patient population used for the LVOT‐VTI measures was similar with the normal ICU population of previous studies.^18^ In terms of reasons for admittance to ICU, we discovered that it seemed to be more difficult to obtain an ideal view of auto LVOT‐VTI of patients with sepsis shock. Meanwhile, our survey revealed that patients with higher SOFA scores and concentration of lactic acid were less likely to acquire the ideal quality of LVOT‐VTI image. As a result, the greater percentage of severe conditions in average group might be the reason for a bit higher in‐hospital mortality than patient in ideal group (40% vs. 22.6%). This phenomenon was consistent with previous studies that more severe the patient was, the less probably to acquire a perfect or ideal view of bedside echocardiography because of the vast majority of supine position, more mechanically ventilated and worse window for the probe placement.^19^, ^20^, ^21^ Consistent with the more severe illness of patients, the greater amount of LVEDd, LA‐d, and percentage of decreased LV wall motion were detected in average group and this observation was associated with higher incidence of heart injury in critically ill patients. ICU patients with more severe conditions were the target population of hemodynamic management by PoCUS. The difficulty to acquire ideal view of auto LVOT‐VTI for them have highlighted the importance of optimizing the technique, such as the application of auto LVOT‐VTI trending instead of baseline auto LVOT‐VTI in the evaluation of fluid responsive in the future research.^8^, ^19^

Despite the different levels of AI‐based image quality, we found that manual and auto LVOT‐VTI values were similar under both ideal and average view. As absolute values of LVOT‐VTI, Goldman et al.^22^ showed that the range of LVOT‐VTI value in a patient with normal SV and CO was from 17 to 23 cm when his or her heart rate (HR) is within the 55‐95 bpm. When the HR is under 55 bpm, the LVOT‐VTI values must be higher than 18 cm; otherwise, a low systolic volume (SV) and cardiac output (CO) are indicated and when the HR is higher than 95 bpm, LVOT‐VTI values must be lower than 22 cm; otherwise, a high SV and CO are suggested. Despite of heart rates, all the mean values of manual LVOT‐VTI and auto LVOT‐VTI were in the range of expected LVOT‐VTI with normal SV and CO. Taking the different range of heart rate into consideration, we could see that there was no low SV when a patient was bradycardia rhythm (HR < 55) while there was no high SV when a patient was tachycardia rhythm (HR > 95) (Supporting Information 1). In summing up, we could see that 14 of the ICU patients newly admitted were labeled as low SV. Moreover, we found that compared with LVEF [LVEF < 45% (*n* = 4), LVEF > = 45 (*n* = 42)], LVOT‐VTI was more sensitive to diagnose low SV (Supporting Information 2) and this result was consistent with a recent article that LVOT‐VTI might be useful for categorizing a low‐flow preserved ejection fraction (HFpEF) phenotype.^23^ Regarding reliability, if expected physiologic responses range between increments of at least 15% in VTI after an intervention, intra‐ and interobserver variability for the measurement of VTI must be lower than these values, otherwise, the margin of error may exceed the patient's physiologic response. Regarding this point, the reported intra‐ and interobserver variabilities were low among studies ranging between 5% and 11%.^20^, ^21^, ^24^, ^25^, ^26^ The range of δLVOT‐VTI in average view was close to it in the ideal view and both of the ranges were less than 15%. In addition to that, we found that manual and auto LVOT‐VTI had statistically significant agreement in both groups. This indicted that it was possible to achieve dependable auto LVOT‐VTI value in average quality image as well. The auto‐VTI® software in our study has been tested in an animal (piglets) experimental model of hemorrhagic shock and demonstrated a better correlation with the CO obtained by thermodilution when compared with the conventional echocardiography technique.^27^ As mentioned above, the auto‐VTI® software could obtain LVOT‐VTI with less effort and saving time, while obtaining the manual LVOT‐VTI often requires several key strokes and may be time‐consuming.^27^ Owing to the application of auto‐VTI® software, we could improve the accuracy of VTI by maintaining the probe in the same position during few minutes for measuring repeatedly the VTI with accuracy, particularly when using the PLR test.^28^

Previous study showed that automatic LVOT‐VTI measurement was feasible and allowed for quick and accurate measurement of cardiac output in novice operators^15^ but it did not mention the impact of different levels of AI‐based image quality on the accuracy in auto LVOT‐VTI measuring.^15^ Our study first verified that the auto LVOT‐VTI in ideal view was more relevant with the manual LVOT‐VTI than the average view. It suggested that AI‐based technology might provide real‐time guidance among PoCUS trainees who lack expertise to acquire the ideal standard view. Not only AI could provide quality assurance for patient care standards,^29^ but also serves as an important modality for PoCUS training. The use of AI allows less experienced operators to receive visual guidance on proper probe placement and adjustment when scanning with standard views.^30^, ^31^ Studies have demonstrated that the use of AI has allowed operators with no prior ultrasound experience to obtain diagnostic ventricular and pericardial images.^30^, ^31^ Cheema et al.^32^ report the successful use of novel AI‐derived technology deployed in the COVID‐19 ICU by critical care physicians with clinical experience but no formal training in ultrasound to obtain PoCUS cardiac images. The continued development and incorporation of AI in POCUS diagnostics may limit expertise dependence and improve accuracy in non‐experienced users. The appeal of AI continues to expand in point‐of‐care markets with the further miniaturization of technology, improved ease of use, lower system cost, increased portability, and greater access to training.^29^

### 4.1 Limitations

The present study had following limitations. First, our study was done in a single center, raising questions on their external generalizability to influence education policy. We will perform the program in other centers to make sure it is sustainable and feasible even in centers with limited resources. Second, standard bedside echocardiography examinations of ICU patients newly admitted acquired by ICU doctors in 2 h and by expert sonographers in 24 h. Due to the interval time, the condition changes of severe patients might be different. This might lead to inconsistent findings between ICU doctors and expert sonographers. However, our research adopted relatively stable variables as key indexes of parasternal long/short‐axis view and apical four/five‐chamber view to ensure objectivity and repeatability of echocardiography parameters between trained ICU doctors and experts. In order to reduce time and rapid fluid induced variations of LVOT‐VTI, the manual and auto LVOT‐VTI were acquired by ICU doctors at the same time when there was no rapid fluid infusion for the patients. Third, our research had not analyzed the influence of fluid responsiveness (FR) measured by auto LVOT‐VTI. In terms of fluid responsiveness, a further study focused on the auto VTI‐trending after mini and normal fluid challenge in sepsis shock will be carried out in our center.

## 5 CONCLUSIONS

In conclusion, our study found that ICU doctors could achieve the high level of expertise as expert sonographers after 3 months of PoCUS training. In the application of auto LVOT‐VTI, nearly two thirds of the enrolled ICU trainees could obtain the ideal view and one third of them could acquire the average view. Our survey revealed that patients with higher SOFA scores and lactic acid were less likely to acquire the ideal view of LVOT‐VTI. Additionally, we found that manual and auto LVOT‐VTI had statistically significant agreement in both ideal and average groups. Furthermore, our study verified that the auto LVOT‐VTI in ideal view was more relevant with the manual LVOT‐VTI than the average view.

## CONFLICT OF INTEREST

The authors declare that there is no conflict of interest that could be perceived as prejudicing the impartiality of the research reported.

## AUTHOR CONTRIBUTIONS

Shanshan Zhai and Hui Wang conceived the presented idea and contributed equally to this study. Shanshan Zhai and Hui Wang wrote the manuscript with support from Lichao Sun, Feng Huo, Shuang Qiu, Xiaoqing Wu, Junyu Ma and Yina Wu. Bo Zhang contributed to the interpretation of the results. All authors discussed the results and contributed to the final manuscript. All authors provided critical feedback and helped shape the research, analysis and manuscript. Jun Duan supervised the project and those findings of this work.

## Supporting information

Supporting Information 1

Supporting Information 2
