# Supplementary material for: Artificial intelligence (AI) versus expert: A comparison of left ventricular outflow tract velocity time integral (LVOT‐VTI) assessment between ICU doctors and an AI tool
Source: J Appl Clin Med Phys. 2022 Jul 11;23(8):e13724. doi: 10.1002/acm2.13724 (PMC9359021; doi:10.1002/acm2.13724)
Supplement: Supplementary file 1 — Supporting Information 1 [file ACM2-23-e13724-s002.docx]

Supplement.1

| Hear rate(bpm) | LVOT-VTI<= 18 | 18 <=LVOT-VTI<= 22 | LVOT-VTI> 22 |
| --- | --- | --- | --- |
| < 55 | 0 | 0 | 2 |
| 55~95 | 7 | 17 | 10 |
| >95 | 7 | 3 | 0 |
