# Supplementary material for: Artificial intelligence (AI) versus expert: A comparison of left ventricular outflow tract velocity time integral (LVOT‐VTI) assessment between ICU doctors and an AI tool
Source: J Appl Clin Med Phys. 2022 Jul 11;23(8):e13724. doi: 10.1002/acm2.13724 (PMC9359021; doi:10.1002/acm2.13724)
Supplement: Supplementary file 2 — Supporting Information 2 [file ACM2-23-e13724-s001.docx]

Supplement.2

| **Paired Chi2 Test** | | **Manual LVOT-VTI** | | |
| --- | --- | --- | --- | --- |
|  |  | +(VTI <18) | -(VTI>=18) | Total |
| **LVEF** | +(LVEF < 45%) | 3 | 1 | 4 |
|  | -(LVEF >45%) | 11 | 31 | 42 |
|  | Total | 14 | 32 | 46 |
| **Results** | Kappa Test | | McNemar Test | |
|  | Chi2 | p | Chi2 | p |
|  | 2.13 | **0.144** | 21.44 | **0.000** |
| Paired Chi2 test, Kappa Test for the measurement of agreement; McNemar Test for the difference between paired proportions | | | | |
